# Stratification of COPD patients towards personalized medicine: reproduction and formation of clusters

**DOI:** 10.1186/s12931-022-02256-7

**Published:** 2022-12-09

**Authors:** Cathelijne M. van Zelst, Lucas M. A. Goossens, Jan A. Witte, Gert-Jan Braunstahl, Rudi W. Hendriks, Maureen P. M. H. Rutten-van Molken, Johannes C. C. M. in’t Veen

**Affiliations:** 1 Department of Pulmonology, Franciscus Gasthuis en Vlietland, Kleiweg 500, 3045 PM Rotterdam, The Netherlands; 2 Department of Pulmonary Medicine, Erasmus MC, University Medical Center, Rotterdam, The Netherlands; 3 Erasmus School of Health Policy and Management, Erasmus University, Rotterdam, The Netherlands

**Keywords:** COPD, Phenotypes, GOLD classification, ABCD assessment tool, Personalized medicine

## Abstract

**Background:**

The global initiative for chronic obstructive lung disease (GOLD) 2020 emphasizes that there is only a weak correlation between FEV_1_, symptoms and impairment of the health status of patients with chronic obstructive pulmonary disease (COPD). Various studies aimed to identify COPD phenotypes by cluster analyses, but behavioral aspects besides smoking were rarely included.

**Methods:**

The aims of the study were to investigate whether (i) clustering analyses are in line with the classification into GOLD ABCD groups; (ii) clustering according to Burgel et al. (Eur Respir J. 36(3):531–9, 2010) can be reproduced in a real-world COPD cohort; and (iii) addition of new behavioral variables alters the clustering outcome. Principal component and hierarchical cluster analyses were applied to real-world clinical data of COPD patients newly referred to secondary care (n = 155). We investigated if the obtained clusters paralleled GOLD ABCD subgroups and determined the impact of adding several variables, including quality of life (QOL), fatigue, satisfaction relationship, air trapping, steps per day and activities of daily living, on clustering.

**Results:**

Using the appropriate corresponding variables, we identified clusters that largely reflected the GOLD ABCD groups, but we could not reproduce Burgel’s clinical phenotypes. Adding six new variables resulted in the formation of four new clusters that mainly differed from each other in the following parameters: number of steps per day, activities of daily living and QOL.

**Conclusions:**

We could not reproduce previously identified clinical COPD phenotypes in an independent population of COPD patients. Our findings therefore indicate that COPD phenotypes based on cluster analysis may not be a suitable basis for treatment strategies for individual patients.

**Supplementary Information:**

The online version contains supplementary material available at 10.1186/s12931-022-02256-7.

## Introduction

The severity of chronic obstructive pulmonary disease (COPD) is defined by forced expiratory volume in 1 s (FEV_1_), divided into four stages of severity (Global initiative for chronic obstructive lung disease; GOLD I-IV) [1]. Nonetheless, the heterogeneity of the clinical presentation and disease development among patients within the same GOLD stage is substantial [2]. GOLD 2011 introduced the ABCD assessment tool to classify stable COPD patients on the basis of airflow limitation, number of exacerbations per year and questionnaires to measure the severity of symptoms: modified Medical Research Council (mMRC) scale and COPD Assessment Test (CAT) [1]. This approach is more comprehensive than airflow limitation alone, but it is still based on a limited number of variables. In GOLD 2017 a refinement of the ABCD assessment tool was suggested, which separated the spirometric GOLD I-IV grades from the ABCD groups and introduced pharmacotherapy recommendations per ABCD group.

An alternative method facilitating subgroup-specific treatment might be established by the identification of phenotypes on the basis of prognostic, demographic, clinical, pathophysiological or therapeutic characteristics. Han et al. proposed the following definition of phenotypes in the context of COPD: “a single or combination of disease attributes that describe differences between individuals with COPD as they relate to clinically meaningful outcomes” [3].

Phenotyping can be aided by using descriptive statistics, such as cluster analysis to identify separate patient groups according to preselected variables [4]. With regards to these variables, patients within a certain cluster are more similar to each other than to patients in different clusters [5]. The identification of coherent clusters may lead to the recognition of phenotypes, which could be a valuable step towards tailored treatment strategies per subgroup.

Several attempts have been made to develop a useful classification of phenotypes of COPD patients [4]. To be potentially useful in clinical practice, the identity of the defined clusters needs to be confirmed in different, independent cohorts of COPD patients, but to the best of our knowledge such replication studies have not been performed yet. Burgel et al. [6] performed more extensive phenotyping of COPD patients based on the clinical variables age, cumulative smoking, airflow limitation, body mass index (BMI), exacerbations per year, dyspnea, health status and depressive symptoms. Hereby, four clinical phenotypes were defined: phenotype 1 were relatively young subjects (median 58 [IQR 55–63] years old) with predominantly severe to very severe respiratory disease, frequent exacerbations and low BMI; phenotype 2 were older patients (median 68 [IQR 60–74] years old) with mild symptoms; phenotype 3 were younger subjects (median 59 [IQR 50–65] years old) with moderate to severe airflow limitation. In the fourth phenotype older patients (median 72.5 [IQR 67–77] years old) with moderate to severe airflow limitation were included. Compared to phenotype 3, these patients had a higher prevalence of depressive symptoms, higher BMI and more severe dyspnea. Patients with comparable FEV_1_ were assigned to different phenotypes [6]. Longitudinal 2-year follow-up showed that phenotype 2 is associated with a very low risk of mortality and that patients with phenotype 1 had the highest mortality rates and died at a younger age [7].

Moreover, in the current literature, cluster classifications are largely based on clinical variables, while behavioral variables are rarely used. Whereas most attention is drawn to smoking behavior [6, 8], other behavioral aspects such as coping, physical activity and quality of life (QOL) are not included. Nevertheless, these are important variables because they influence the impact of self-management interventions and can interfere with active participation [9]. For example, a high rate of physical activity is known to increase shortness of breath and therefore it is avoided by most COPD patients. On the other hand, in the long run physical exercise in COPD is associated with a reduction of shortness of breath [10]. Shortness of breath during physical activity can be mechanically influenced by air trapping, which makes this an interesting physiological parameter to add to phenotyping [11].

The aim of this study is threefold. First, to investigate whether the results of our cluster analyses match the ABCD groups defined by the GOLD criteria, which are either based on CAT and exacerbation frequency or on mMRC and exacerbation frequency. Second, to address whether the four COPD phenotypes previously identified by Burgel et al. can be reproduced by cluster analysis in another real-world COPD cohort. Third, to determine whether the addition of six new variables: QOL, fatigue, satisfaction relationship, air trapping and steps per day and activities of daily living improves the classification into distinct subgroups.

## Methods

### Study design

We performed three independent cluster analyses of COPD patient characteristics. In the first analysis, we aimed to identify clusters that corresponded with the ABCD groups [1]. Secondly, we investigated the reproducibility of Burgel’s clusters in our study population [6]. Thirdly, we added six new variables, QOL, fatigue, satisfaction relationship, air trapping and steps per day and activities of daily living to the parameter of the second analysis.

### Setting and participants

Data were part of a registry study of patients with asthma and COPD, who were newly referred to the Franciscus Gasthuis and Vlietland Hospital in Rotterdam, the Netherlands, a center of excellence for asthma and COPD. All referred COPD patients (n = 155) who completed a previously published [12] comprehensive assessment during the period December 2012 till December 2017 were included. The diagnosis of COPD was based on an assessment by a pulmonologist and confirmed by spirometry (FEV1 / forced vital capacity (FVC) < 0.7). In this study, we used pseudonymized assessment data. Ethics approval for this study was waived by the Institutional Research Board of the Franciscus Gasthuis & Vlietland, Rotterdam, the Netherlands, because routinely collected health care data were used after pseudonymization.

### Data collection

The following variables were collected for all patients:

*Lung function.* FEV_1_, FVC, and static and dynamic hyperinflation were performed according to the ATS/ ERS taskforce “standardization of spirometry” [13, 14]. Values for post-bronchodilation dynamic hyperinflation were measured by metronome-paced tachypnea after bronchodilation (400 µg of inhaled salbutamol) [15]. Lower levels of air trapping (dynamic hyperinflation after bronchodilation measured in liters decreasing inspiratory capacity) reflect poor outcome. All tests were performed with the Vmax Sensor Medics Viasys, type 6200 Encore.

*Pack years.* A pack year is defined as twenty cigarettes smoked per day for 1 year.

*Body mass index (BMI):* BMI is defined as the body mass divided by the square of the body height, expressed in units kg/m^2^ [16].

*Exacerbations.* The number of antibiotic courses and/or systemic steroids for their respiratory disease in the previous year (0, 1, 2 or 3 +).

*Symptoms and health status.* Modified Medical Research Council (mMRC) is a five-item questionnaire to score the dyspnea of COPD patients [17, 18] (Table 1). The Clinical COPD Questionnaire (CCQ) is a ten-item questionnaire about symptom severity [19]. A higher score indicates a worse health status. The minimal clinically important difference is 0.4 [20]. The Beck Depression Inventory for primary care (BDI-PC) was used to score symptoms of depression independently of physical function [21, 22].

**Table 1:** Scoring range of questionnaires

Variables	Scoring range*
mMRC dyspnoea score	1–5
CCQ total score	0–6
BDI-PC total score	0–21
BOD score	0–7
NCSI quality of life	1–101.6
NCSI satisfaction relationship	2–10
NCSI activities of daily living	0–135.5
NCSI fatigue	8–56

*Higher scores reflect poor condition

*BOD-score.* BOD-score includes the variables BMI, airflow obstruction and dyspnea in COPD (Table 1). Higher BOD scores for indicate a greater risk of death [23].

*Physical activity.* Physical activity was measured by an activity tracker (McRoberts© Triaxial accelerometer) during 1 week. The mean number of steps in 24 h over 7 days was used for analyses.

*ABCD groups.* Group ‘A’ includes patients with mMRC 0–1 or COPD Assessment Test (CAT) < 10 and 0–1 exacerbation per year; group ‘B’ includes patients with mMRC ≥ 2 or CAT ≥ 10 and 0–1 exacerbation per year; group ‘C’ includes patients with ≥ 2 exacerbations or ≥ 1 exacerbation leading to hospital admission with mMRC 0–1 or CAT < 10 and group ‘D’ includes patients with ≥ 2 exacerbations or ≥ 1 exacerbation leading to hospital admission with mMRC ≥ 2 or CAT ≥ 10 [1].

*Nijmegen Clinical Screening Instrument (NCSI).* Four NCSI domains were included; QOL, satisfaction relationship, behavioral impairment (termed activities of daily living) and fatigue [24]. The minimum and maximum scores are shown in Table 1.

### Analyses

Following the methodology applied by Burgel et al. [6], we used Principal Component Analysis (PCA) and Ward’s hierarchical cluster analysis. In Ward’s method, the analysis starts with each subject forming its own cluster [25]. Step by step, the number of clusters is reduced until all subjects are in one cluster. In each step, the two most similar clusters from the previous steps are combined, based on the variables that are have been selected to describe the clusters. These two clusters are selected in such a way that the total of the variances of all variables within the new clusters is as small as possible. Before clusters were formed, PCA was used to reduce the number of variables by replacing them by newly created uncorrelated variables (‘components’) with minimal information loss [26].

Dendrograms were used to graphically represent the hierarchical relationship between the clusters and the distance between them. The resulting cluster solutions were described and compared to determine the optimal number of clusters. All statistical analyses were performed using Stata/SE 15.1. Following Burgel et al. [6], variables were standardized (i.e. using Z-score or PCA) before they were included in the cluster analysis and categorical or dichotomous variables were expressed numerically. PCA were performed to reduce interaction between the variables included in the cluster analyses. Components with an eigenvalue > 1 were used.

In the first cluster analysis, in which we aimed to match ABCD groups, two times two variables (exacerbations per year and mMRC versus exacerbations per year and CAT) were used. We used two types of symptom questionnaires (CAT and mMRC) as a marker of disease burden, because GOLD uses either one of these to form the ABCD groups.

In the second cluster analysis, in which we investigated whether the four clinical phenotypes of Burgel could be reproduced, the following eight variables were used: age, packyears, FEV_1_, BMI, mMRC, CCQ, BDI-PC and the number of exacerbations per year. Given the availability of data, CCQ analysis was used instead of the St. George Respiratory Questionnaire [27], and BDI-PC instead of the Hospital Anxiety and Depression Scale [28]. For comparative purposes in Fig. 3, we projected the CCQ and BDI-PC scores of our study onto the SGRQ and HADS (Burgel’s study) as follows. We divided the 50th, 25th and 75th percentiles of the CCQ and BDI-PC by the score range (which is 6 and 21 respectively), and multiplied this with the score range of the SGRQ and HADS which is 100 and 42 respectively).

**Fig. 1:**
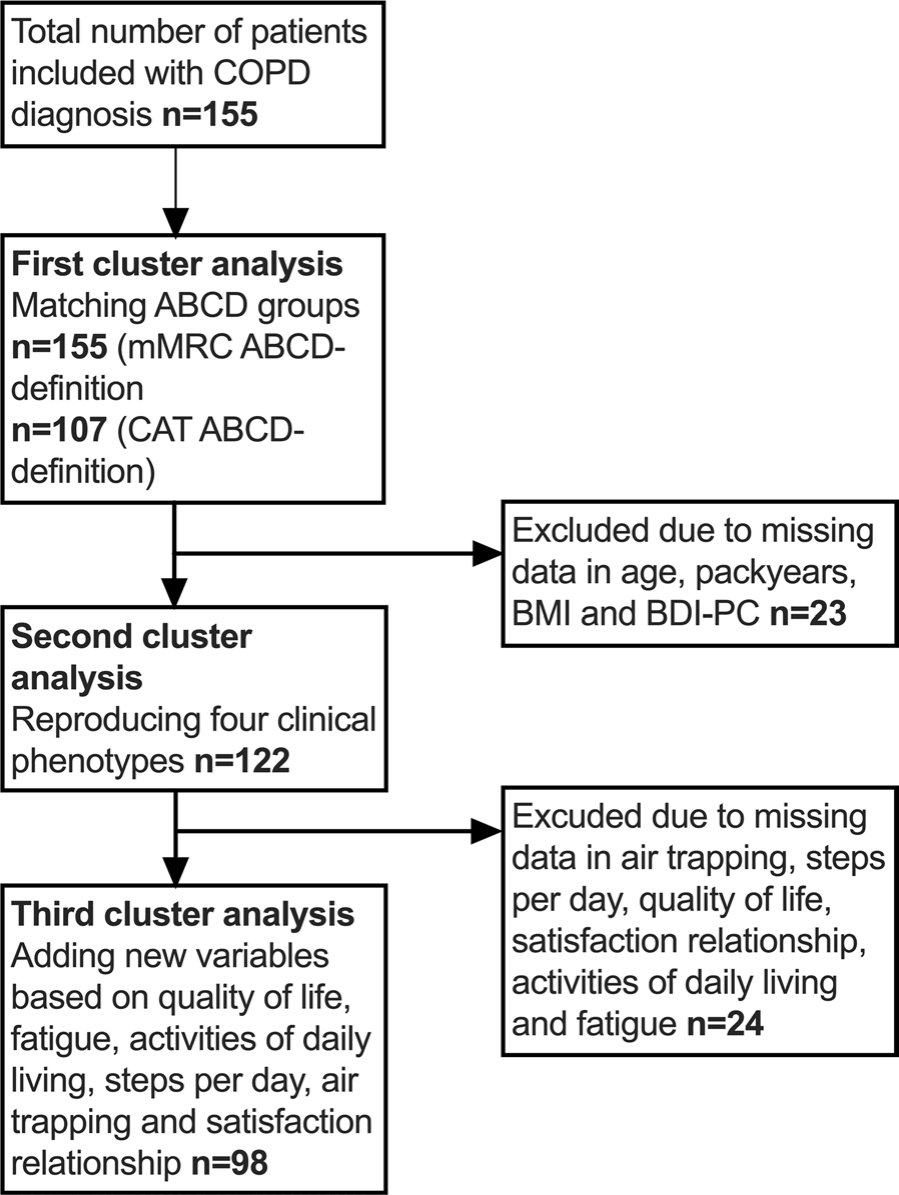
Flowchart of patient enrollment in the different cluster analyses

In the third cluster analysis, six new variables, NCSI QOL, NCSI satisfaction relationship, NCSI fatigue, air trapping, steps per day and NCSI activities of daily living were added (Fig. 1).

## Results

### Patient characteristics and ABCD classification

COPD Patients were divided into ABCD groups, based on the mMRC definition (n = 155) or the CAT definition (n = 107) (Shown in Table 2 and Additional file 1: Table S1, respectively).

**Table 2:** Cluster analysis (ABCD groups) using the variables exacerbation number and mMRC

Cluster	Group A	Group B	Group C	Group D
Number	61	37	31	26
*Cluster number*				
1	0*	0	24 (65)	13 (35)
2	0	9 (41)	0	13 (59)
3	14 (33)	28 (67)	0	0
4	47 (87)	0	7 (13)	0
Variables used in clustering**				
Exacerbations p/y	0 [0–1]	1 [0–1]	2 [2–3]	3 [3–3]
mMRC dyspnoea score	1 [0–1]	2 [2–3]	1 [0–1]	3 [2–4]
*Other patient and disease characteristics*				
Male/ female %	53/47	62/38	45/55	42/58
Age in years	62 [54–68]	66 [60–71]	62 [55–67]	61 [52–70]
Smoked PY	40 [21–50]	40 [25–63]	28 [19–50]	33 [26–55]
FEV1% pred	62 [48–73]	52 [43–65]	63 [43–69]	54 [30–70]
GOLD stage %				
1	15	11	23	12
2	59	50	52	44
3	26	33	22	20
4	0	6	3	24
BMI kg/m^2^	26 [21–30]	31 [22–36]	27 [24–29]	24 [20–30]
CCQ total score	1.4 [0.8–2.5]	2.7 [1.9–3.3]	2.1 [1.6–2.7]	3.2 [2.7–3.9]
CAT	13 [9–18]	21 [16–24]	18 [13–23]	27 [21–28]
BDI-PC total score	1 [1–2]	1 [1–4]	1 [0–4]	3 [2–6]
BOD score	1 [1–3]	4 [3–5]	2 [1–3]	5 [4–7]
Steps per day	5261 [3863–8260]	4042 [2466–5733]	5743 [4473–6904]	3936 [2226–5777]
NCSI quality of life	13 [7–21]	20 [11–28]	14 [6–28]	32 [22–42]
NCSI satisfaction relationship	3 [2–4]	3 [2–5]	2 [2–4]	5 [3–6]
NCSI activities of daily living	8 [3–17]	22 [14–27]	12 [5–24]	28 [13–40]
NCSI fatigue	35 [28–41]	42 [38–48]	43 [35–49]	44 [38–51]

*Data are presented as N (%) or median [25–75 interquartile], unless otherwise stated. *PY* packyears, *FEV1% pred* forced expiratory volume in 1 s percentage predicted, *BMI* Body Mass Index, *mMRC* Modified Medical Research Council, *CCQ* Clinical COPD Questionnaire, *BDI-PC* Beck Depression Inventory for primary care. *Definition of group A: mMRC < 2 and exacerbations < 2, group B: mMRC >  = 2 and exacerbations < 2, group C: mMRC < 2 and exacerbations >  = 2, group D: mMRC >  = 2 and exacerbations >  = 2

**Hierarchical clustering is performed based on two variables: Exacerbation per/year and mMRC

Using the mMRC-based classification, patients in group A (n = 61) were ~ 53% male with a median age of ~ 62y [IQR 54–68y] and scored best on CCQ total score, BOD score, QOL, activities of daily living and fatigue. Patients in group B (n = 37) were ~ 62% male with a median age of ~ 66y [IQR 60–71y]. All four GOLD stages of airflow limitation were represented in group B, ~ 11% of the patients were classified in GOLD stage I and ~ 6% in GOLD stage IV. In group C (n = 31), the patients were ~ 55% female with a median age of ~ 62y [IQR 55–67y]. They had the lowest number of smoked pack years (PY) with a median value of 28 [IQR 19–50 PY], lowest depression score and most steps per day (median value 5743 [IQR 4473–6904]). Patients in group D were ~ 58% female with a median age of ~ 61y [IQR 52–70y]. All four GOLD stages of airflow limitation were represented in group D with ~ 12% GOLD stage I and ~ 24% stage IV. They scored worst on CCQ, depressions score, BOD score, QOL, activities of daily living and fatigue.

Using the CAT-based definition, patients in group A (n = 15) were ~ 60% female with a median age of ~ 63 [IQR 59–68y]. They had the lowest FEV_1_ with a median value ~ 49% of predicted [IQR 42–66] and only GOLD stage II and III were represented, resp. ~ 53% and ~ 47%. Patients in group B (n = 51) were ~ 67% male with a median age of ~ 64 [56–68y]. They scored worst on activities of daily living, QOL, satisfaction relationship and had the highest number of PY with a median value of ~ 62 [IQR 51–74 PY]. The four GOLD stages of airflow limitation were represented in group B with ~ 18% GOLD stage I and ~ 4% stage IV. Patients in group C (n = 2) included one male and one female, both GOLD stage II. Patients in group D (n = 39) were ~ 54% female with a median age of ~ 62y [IQR 55–69y] and scored worst on CCQ total score, depression score and fatigue. The four GOLD stages of airflow limitation were represented in group D with ~ 21% GOLD stage I and ~ 15% stage IV.

### First cluster analysis: relation to ABCD groups

We performed cluster analysis based on exacerbation numbers in conjunction with mMRC (n = 155 patients) or CAT values (available in n = 107 patients).

**Fig. 2:**
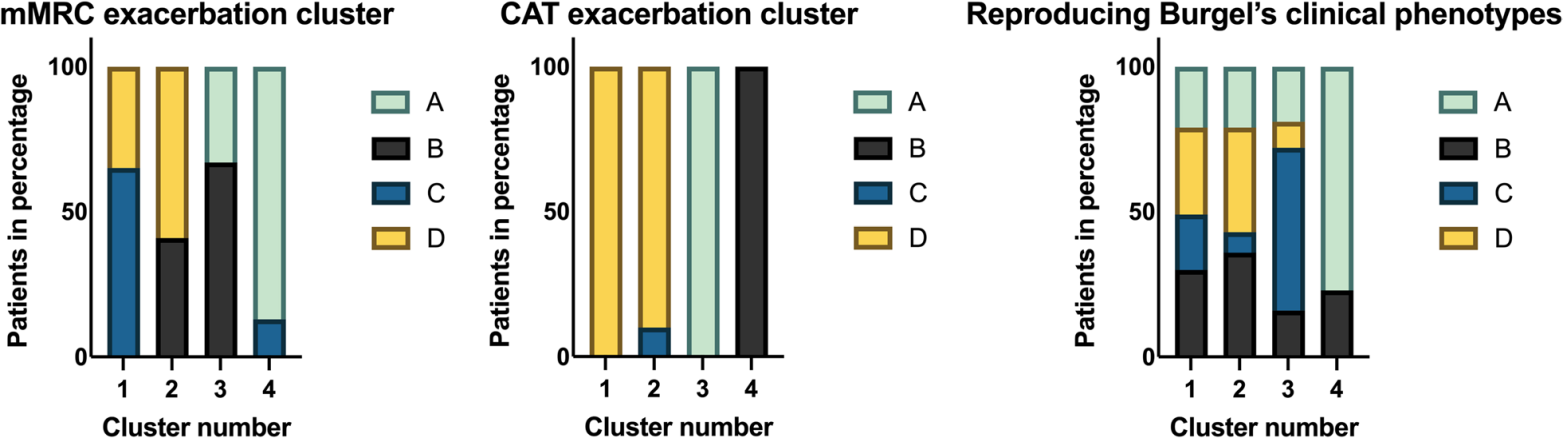
Overview of the cluster analyses categorized in ABCD groups. The three different cluster analyses (resp. used variables: mMRC and exacerbation frequency, CAT and exacerbation frequency and Burgels eight clinical variables) are shown categorized in group A, B, C and D. The definition of the ABCD group in the reproduction phenotype is based on mMRC. Definition ABCD groups: Group ‘A’ includes patients with mMRC 0–1 or CAT < 10 and 0–1 exacerbation per year; group ‘B’ includes patients with mMRC ≥ 2 or CAT ≥ 10 and 0–1 exacerbation per year; group ‘C’ includes patients with ≥ 2 exacerbations or ≥ 1 exacerbation leading to hospital admission with mMRC 0–1 or CAT < 10 and group ‘D’ includes patients with ≥ 2 exacerbations or ≥ 1 exacerbation leading to hospital admission with mMRC ≥ 2 or CAT ≥ 10; mMRC: modified Medical Research Council; CAT: COPD Assessment Test

Using the mMRC scale questionnaires, four clusters (n = 37, n = 22, n = 42, and n = 54) were identified, which showed only limited parallels with the ABCD groups (Table 2; Fig. 2). For the individual clusters, the largest contributing fraction of one of the ABCD groups was ~ 59–87%. By contrast, the four CAT-based clusters displayed a high level of similarity with the ABCD groups: in each of the four clusters ~ 90–100% of the patients were classified as a single ABCD group (Fig. 2, Additional file 1: Table S1): in clusters 1, 3 and 4 all patients fit in group D (CAT ≥ 10 and exacerbations ≥ 2), group A (CAT < 10 and exacerbations < 2) and group B (CAT ≥ 10 and exacerbations < 2), respectively. Only for cluster 2 we found patients classified in two different ABCD groups: group D (~ 90%) and group C (~ 10%; (CAT < 10 and exacerbations ≥ 2).

### Second cluster analysis: reproducing four clinical phenotypes

In the second analysis we aimed to reproduce Burgel’s four clinical phenotypes and included 122 COPD patients from whom a complete set of the preselected variables (see Materials and Methods), was available (Fig. 1).

The PCA transformed the eight original variables (see Additional file 1: Table S2) into independent components, the first four of which contained ~ 71% of the information and had an eigenvalue > 1, which indicated that they contained more information than the average of the replaced variables. To PC1 the variables CCQ total score, BDI-PC, mMRC and exacerbations contributed the most. PC2 was predominantly based on age and BMI. PC3 reflected FEV1%pred and BMI and was independent of mMRC and number of exacerbations. Finally, PC4 was correlated with numbers of exacerbation and inversely correlated with smoking pack years and BDI-PC (see Additional file 1: Table S2).

Based on these four components, the four-cluster solution consisted of the following clusters: reproduction cluster 1 (n = 37) with ~ 54% female and median age of ~ 64y [IQR 57–71y]. These patients had the lowest FEV1% predicted ~ 39 [IQR 30–52] and lowest value of BMI ~ 21 [IQR 19–25]; reproduction cluster 2 (n = 14) with 71% male and median age ~ 55 [IQR 48–61], had the highest number of PY ~ 50 [IQR 30–66] and scored worst on depression score, CCQ and QOL. Reproduction cluster 3 (n = 32) was ~ 53% female with median age ~ 65 [58–72]. They had the lowest number of PY: ~ 22 [IQR 5–35] and the most exacerbations per year ~ 2 [IQR 1–3 PY]. Reproduction cluster 4 (n = 39) with ~ 59% male and median age ~ 63 [57–68], scored best on mMRC, CCQ, exacerbations per year, depression score and activities of daily living.

**Fig. 3:**
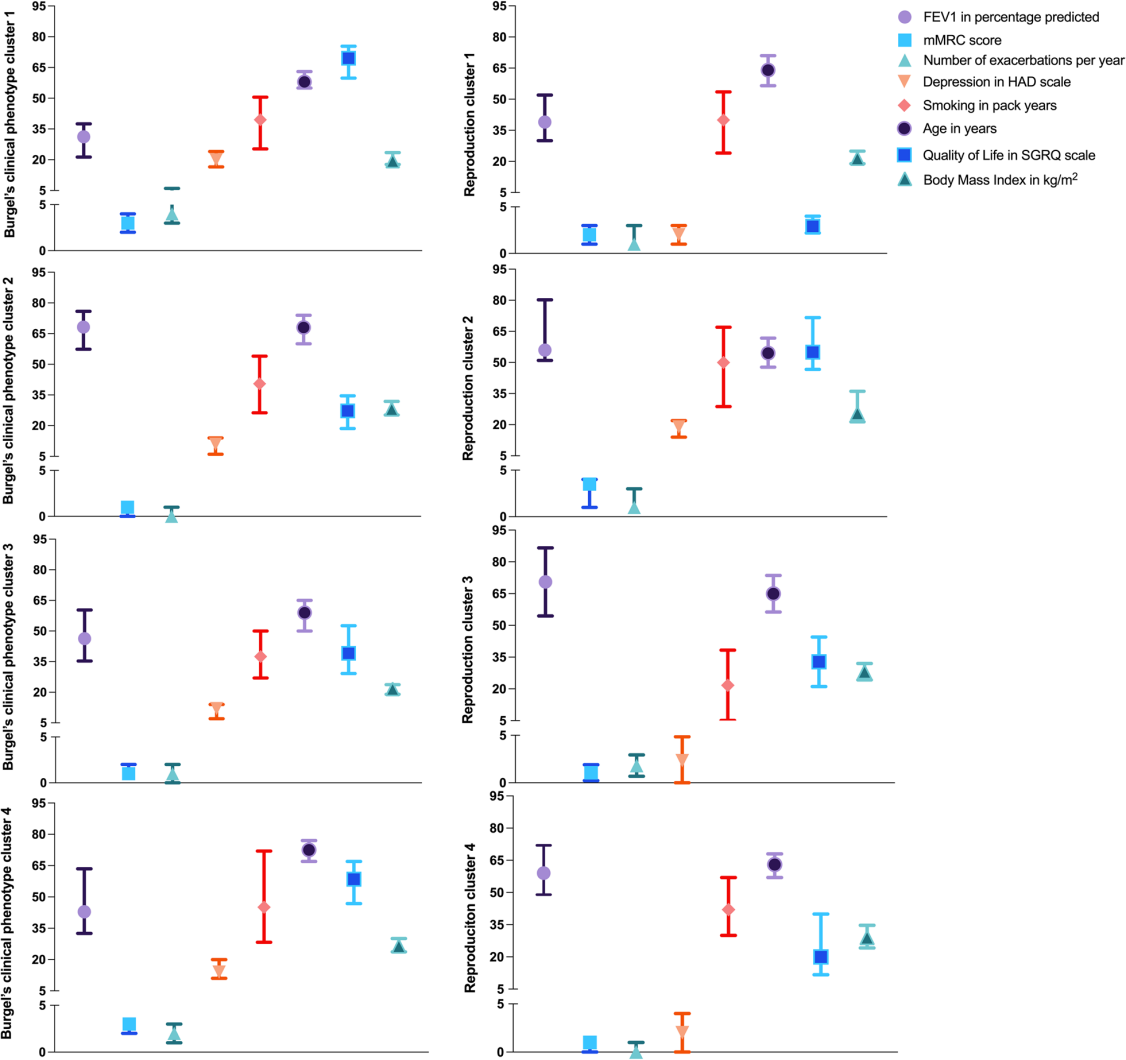
Overview of cluster analysis replicating Burgel. In the first column Burgel’s four clinical phenotypes are shown, and in the second column our four reproduction clusters are visualized. Burgel used Hospital Anxiety and Depression (HAD) scale as depression scale whereas the reproduction cluster used the Beck Depression Inventory-Primary Care (BDI-PC) scale. Burgel used St. George’s Respiratory Questionnaire (SGRQ) to measure quality of life (QOL) and the reproduction cluster used CCQ. To improve optical comparison between the two cohorts, the BDI-PC median [IQR] of the replication clusters are re-calculated in the range of the HAD scale and the CCQ median [IQR] re-calculated in the range of the SGRQ. Re-calculation is outlined in the Methods section

Only one of our four clusters was comparable with one of the phenotypes of Burgel: reproduction cluster three appeared to be similar to phenotype two of Burgel et al. [6]. The patient groups had a similar median age (~ 65y [IQR 58–72y] vs. 68y [IQR 60–74y]), mMRC score (1 [IQR 0.5–1.5] vs. 1 [0–1]), and BMI value (28 [25–31] vs 28.1 [25.2–31.9]). None of the three other clusters were comparable with the phenotypes of Burgel et al. All levels of mMRC and BOD scores were present across our groups. Our clusters were mainly separated by PY, BMI and depression scale (Table 3; Fig. 3). None of the four clusters we identified matched with any of the ABCD groups (Fig. 2).

**Table 3:** Cluster analysis reproducing four clinical phenotypes

	Reproduction cluster 1	Reproduction cluster 2	Reproduction cluster 3	Reproduction cluster 4
Number	37	14	32	39
Variables used in clustering*				
Age in years	64 [57–71]**	55 [48–61]	65 [58–71.5]	63 [57–68]
Smoked PY	40 [25–53]	50 [30–66]	22 [5–35]	42 [30–57]
FEV1% pred	39 [30–52]	56 [51–79]	68 [62–80]	59 [49–72]
BMI kg/m^2^	21 [19–25]	26 [21–35]	28 [25–31]	29 [24–35]
mMRC dyspnoea score	2 [1–3]	3.5 [1–4]	1 [0.5–1.5]	1 [0–1]
CCQ total score	2.5 [2–3.2]	3.3 [2.8–4.3]	1.9 [1.3–2.7]	1.2 [0.7–2.4]
BDI-PC total score	1 [1–4]	9.5 [7–11]	1 [0–2.5]	1 [0–2]
Exacerbations p/y	1 [1–3]	1 [1–3]	2 [1–3]	0 [0–1]
*Other patient and disease characteristics*				
Male/female %	46/54	71/29	47/53	59/41
FVC% pred	87 [72–101]	89 [77–105]	99 [91–115]	93 [75–108]
GOLD stage %				
1	0	21	25	10
2	35	64	66	64
3	46	7	9	26
4	19	7	0	0
BOD score	4 [4–5]	5 [1–5]	2 [1–2]	2 [1–3]
Steps per day	5283 [3165–7116]	3032 [2277–3936]	5037 3416–6797]	5041 [3371–8371]
NCSI quality of life	19 [11–31]	57 [41–63]	14 [6–24]	12 [6–23]
NCSI satisfaction relationship	3 [2–5]	6 [4–7]	2 [2–3]	3 [2–4]
NCSI activities of daily living	22 [12–30]	18 [9–28]	17 [5–29]	12 [3–18]
NCSI shortness of breath	12 [10–17]	16 [12–20]	10 [8–15]	10 [7–13]
NCSI fatigue	42 [37–47]	49 [38–55]	39 [32–50]	38 [29–45]
*Main corresponding ABCD groups*				
A	8 (21)	3 (21)	6 (19)	30 (77)
B	11 (30)	5 (36)	5 (16)	9 (23)
C	7 (19)	1 (7)	18 (56)	0 (0)
D	11 (30)	5 (36)	3 (9)	0 (0)

*Hierarchical clustering is performed based on PCA of eight variables: age, packyears, FEV_1_% pred, BMI, mMRC, CCQ, BDI-PC, exacerbation per year

**Data are presented as N (%) or median [25–75 interquartile], unless otherwise stated. *PY* packyears, *FEV1% pred* Forced Expiratory Volume in 1 s percentage predicted, *FVC* Forced Vital Capacity, *BMI* Body Mass Index, *MMRC* Modified Medical Research Council, *CCQ* Clinical COPD Questionnaire, *BDI-PC* Beck Depression Inventory for primary care. Definition of group A: mMRC < 2 and exacerbations < 2, group B: mMRC >  = 2 and exacerbations < 2, group C: mMRC < 2 and exacerbations >  = 2, group D: mMRC >  = 2 and exacerbations >  = 2

### Third cluster analysis: addition of six new variables based on behaviour

**Fig. 4:**
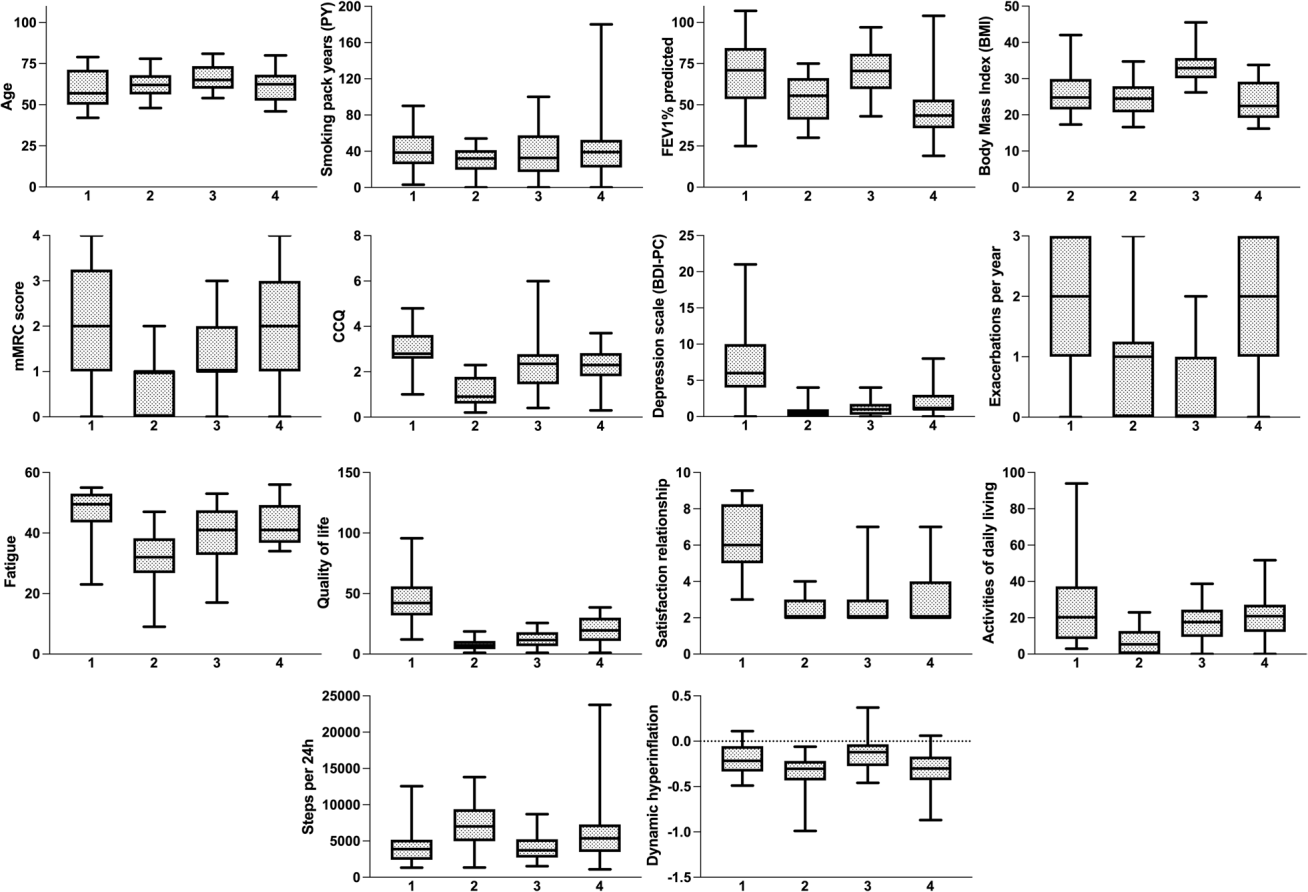
Overview of cluster analysis behavioral variables included. Six new variables; NCSI QOL, NCSI satisfaction relationship, activities of daily living, fatigue, air trapping and steps per day were added to the eight clinical variables of Burgel. These added variables altered the clusters substantially and led to the formation of four clusters that mainly differed from each other on non-physiological parameters. The four behavioral clusters are represented in the X-axis

In the third cluster analysis, 98 patients with COPD were selected for which 14 variables were available: in addition to the eight variables described above (Table 2) we included the following six variables: QOL, fatigue, satisfaction relationship, air trapping, steps per day, and activities of daily living (Fig. 1). The first five components of the PCA contained ~ 68.5% of the information (eigenvalue > 1). Correlations between these five components and the fourteen variables are shown in Additional file 1: Table S3. Second, Ward’s cluster analysis was performed, resulting in the identification of four new clusters (n = 22, n = 26, n = 24, n = 26) that mainly differed from each other in the number of steps per day, NCSI QOL and NCSI activities of daily living (Table 4, Fig. 4). The variables age, PY, mMRC and NCSI satisfaction relationship and NCSI fatigue largely overlapped across the four clusters. Cluster 1 was defined by worse scores on NCSI QOL, NCSI satisfaction relationship, NCSI activities of daily living and NCSI fatigue. Cluster 2 was defined by the best scores on CCQ, depression scale, NCSI QOL, NCSI activities of daily living and most median steps per day 7010 [IQR 5063–9343]. Cluster 3 was characterized by the highest BMI values (33 [IQR 30–36]), low levels of NCSI QOL 11.5 [6.7–17.9] but the lowest levels of dynamic hyperinflation measured in liters decreasing inspiratory capacity after bronchodilation (− 0.12 [IQR − 0.27 to − 0.04L]). Cluster 4 was defined by the highest level of NCSI activities of daily living (20.9 [12.3–26.0]). The new clusters with extra variables were not comparable to the clusters from the second cluster analysis reproducing clinical phenotypes.

**Table 4:** Cluster analysis behavior variables included

Cluster	1	2	3	4
Number	22	26	24	26
Variables used in clustering*				
Age in years	57 [50–71]**	62 [57–68]	65 [60.5–73]	63 [53–68]
Smoked PY	39 [26–57]	32 [20–40]	33 [19–55]	39 [23–50]
FEV1% pred	71 [54–84]	56 [42–66]	71 [60–80]	44 [36–53]
Dynamic hyperinflation post [air trapping]	− 0.22 [− 0.31–0.06]	− 0.31 [− 0.41–0.22]	− 0.12 [− 0.27 to − 0.04]	− 0.3 [− 0.43 to − 0.17]
BMI kg/m^2^	25 [23–29]	25 [21–28]	33 [30–36]	22 [19–29]
MMRC dyspnoea score	2 [1–3]	1 [0–1]	1 [1–2]	2 [1–3]
CCQ total score	2.8 [2.6–3.6]	0.9 [0.6–1.7]	2.4 [1.5–2.8]	2.3 [1.8–2.8]
BDI-PC total score	6 [4–10]	0.5 [0–1]	1 [0.5–1.5]	1 [1–3]
Exacerbations p/y	2 [1–3]	0.5 [0–1]	1 [0.5–1.5]	1 [1–3]
Steps per day	3885 [2442–5147]	7010 [5063–9343]	3727.5 [2781–5066]	5361 [3516–7221]
NCSI quality of life	42.1 [32.1–55.6]	7.4 [4.4–10.4]	11.5 [6.7–17.9]	19.6 [11–29.7]
NCSI satisfaction relationship	6 [5–8]	2 [2–3]	2 [2–3]	2 [2–4]
NCSI activities of daily living	20.3 [8.8–36.9]	5.4 [0–12.7]	17.6 [10.5–23.5]	20.9 [12.3–26.0]
NCSI fatigue	49.5 [44–53]	32 [27–38]	41 [33.5–47]	41 [37–49]
*Other patient and disease characteristics*				
Male/ female %	55/45	38/62	67/33	46/54
FVC% pred	0.895 [0.83–1.05]	1.03 [0.87–1.11]	0.9 [0.84–1.01]	0.96 [0.76–1.04]
GOLD stage %				
1	27	0	25	7
2	59	62	67	31
3	9	38	8	46
4	4	0	0	15
BOD score	2 [1–5]	2 [1–3]	2 [1–3]	4 [3–5]

*Hierarchical clustering is performed based 5 components with an eigenvalue > 1 with variables: age, packyears, FEV_1_% pred, BMI, mMRC, CCQ, BDI-PC, number of exacerbations per year, steps per day, QoL, satisfaction relationship, activities of daily living, fatigue, and dynamic hyperinflation

**Data are presented as N (%) or median [25–75 interquartile], unless otherwise stated. *PY* packyears, *FEV1% pred* Forced Expiratory Volume in 1 s percentage predicted, *FVC* Forced Vital Capacity, *BMI* Body Mass Index, *MMRC* Modified Medical Research Council, *CCQ* Clinical COPD Questionnaire, *BDI-PC* Beck Depression Inventory for primary care. *BOD* Body mass index, airflow Obstruction and Dyspnea score

## Discussion

In a real-world COPD cohort, we were essentially able to identify the GOLD ABCD groups based on CAT, but only one of the four clinical phenotypes described by Burgel et al. [6]. The addition of the six new variables QOL, fatigue, satisfaction relationship, air trapping, steps per day and activities of daily living to the clinical variables, resulted in the formation of four new clusters that did not match the original clinical phenotypes. The new clusters mainly differed in QOL and physical activity, while the previously formed clusters based on clinical variables were very heterogeneous in satisfaction relationship, fatigue, QOL, air trapping, steps per day and activities of daily living.

Burgel et al. suggested it is important to apply PCA and cluster methodology to other COPD cohorts to examine whether similar, or different COPD phenotypes can be identified in different populations [6]. In our cohort, we first attempted to reproduce clinical COPD phenotypes before we added new variables. At first, we clustered based on ABCD GOLD criteria. We noticed that CAT provided a better prediction of the ABCD groups than mMRC, which may be explained by the fact that CAT is a more extensive questionnaire for dyspnea. Second, when we aimed to reproduce the clinical COPD phenotypes as identified by Burgel et al. [6], our clusters were mostly separated by PY, BMI and depression scale. These variables are very different from those that differentiated the original clusters in the report by Burgel et al. (age, airflow limitation and symptoms). It remains unclear why we could not reproduce the clusters of Burgel et al., in our study. Possible explanations include (i) unmeasured confounding factors, (ii) greater heterogeneity in the COPD population, or (iii) the use of slightly different questionnaires.

Not only Burgel et al. used cluster analysis to identify clinical COPD phenotypes. Cluster analysis was previously applied to predict the first acute COPD exacerbation [29]; clusters were identified on the basis of lung function assessment [30] and comorbidity clusters related to inflammatory markers were formed [31]. Although these studies all use similar cluster methods, the clinical variables that were used differed. Clustering on exacerbation type or comorbidities can have clinical value when the clusters are reproducible or correlate with applicable longitudinal data. A longitudinal study based on comorbidity clusters [31] was performed, to associate the changes in exercise performance and health status after pulmonary rehabilitation [32]. This study showed that none of the comorbidity clusters influence the likelihood of clinically meaningful change in exercise performance and health status following pulmonary rehabilitation. The authors conclude that comorbidities in COPD patients should not preclude patients from following pulmonary rehabilitation. Clustering on lung function assessment resulted in seven different clusters [30]. However, based on health status these clusters could not be differentiated from each other because of small differences in mMRC and CCQ [30]. These cluster analyses illustrate the heterogeneity across individual COPD cohorts and the complexity of the identification of COPD phenotypes. In another study the variables used were comparable with our study; COPD phenotypes were clustered according to levels of physical activity, body composition, health related quality of life (HRQoL) and sedentary behavior [33]. Three groups were identified. Phenotype 1 was more physically active and less sedentary compared with phenotype 2 and 3. Phenotype 2 was older and phenotype 3 had worse HRQoL and body composition. Lung function did not differ across the three phenotypes. These results are in line with our behavioral clustering results **(**Table 2). However, inclusion of these variables in the cluster analysis changed the previously formed clusters based on the clinical variables of Burgel (Table 3), which demonstrates that these clusters are not stable. A study in a COPD population discriminating on asthma, emphysema and chronic bronchitis symptoms, the main phenotypes were recognized by easy to obtain clinical characteristics such as smoke exposure and questionnaires on complaints [34]. In parallel to Burgel’s study, we also excluded patients diagnosed with asthma-COPD overlap syndrome, in order to prevent clustering based on smoke exposure and symptom severity. We used the study of Burgel et al. as a reference to reproduce clinical phenotypes, because of a good matching with our clinical variables and because these phenotypes focus on treatable traits instead of future risk factors [35].

The first strength of the study is that we used the same method to form the hierarchical clusters as Burgel did. Second, as we did not use specific exclusion criteria for our real-world COPD patient cohort, our results are expected to have a good external validity for patients in secondary care. All patients with COPD were diagnosed by a pulmonologist. The data we collected were routinely available in daily practice.

Some limitations need to be mentioned. It remains challenging to directly compare our clustering analysis to the clustering by Burgel. First, although the cluster methodology was identical, we did not have exactly the same variables as Burgel et al. We used two different questionnaires: the Clinical COPD Questionnaire (CCQ) instead of the St. George Respiratory Questionnaire (SGRQ), and the Beck Depression Inventory (BDI-PC) instead of the Hospital Anxiety and Depression Scale (HADS). However, the two questionnaires to measure depression—HADS and BDI-PC—are highly correlated [21, 28]. The CCQ and SGRQ are disease-specific questionnaires that measure shortness of breath, amongst others, and are also highly correlated [19, 27, 36]. Compared to the CCQ, the SGRQ is more extensive and includes QOL-related questions. It is possible that these differences in the questionnaires explain the inability to reproduce the clinical clusters, however we would expect more similarity between the clusters because the residual six variables were identical. A second limitation is the small sample size. In the second analysis, 8 variables were used for clustering in a sample of 122 patients, compared to 322 patients in the study of Burgel et al. [6]. Only patients with a complete set of variables could be included in our cluster analyses, which resulted in a small sample size. Phenotyping based on cluster analysis may improve when the number of included patients increase or more suitable variables are added. Third, there may be critical differences between the two cohorts. We included ~ 48% females, whilst Burgel et al. included ~ 23% females. Perez et al. showed that female COPD patients are younger, have lower pack-years, higher FEV1%, lower BMI and exacerbate more often [37]. Moreover, the clinical characteristics of the population of COPD patients may well differ across different medical centers. Newly formed clinical phenotypes need longitudinal validation to determine how they are associated with important clinical outcomes of disease progression or mortality, before conclusions on their clinical relevance can be drawn [3].

In our study, clinical characteristics were used in an attempt to identify clusters as a step towards tailored treatment strategies per subgroup of COPD patients. The inability to reproduce earlier reported clusters in our real-world COPD population questions the relevance of clustering approaches for clinical practice. Clinical practice seems to call for personalized medicine [38], given the heterogeneity of the COPD population even within clusters. Individual patient characteristics should be the main focus to improve clinical outcomes and minimize unnecessary side effects for individual patients with COPD [39]. In this context, it may be more productive to develop personalized medicine approaches based on treatable traits [40, 41], than on clinical phenotypic characteristics.

## Conclusion

In this study we used statistical cluster analyses in a real-world COPD cohort to identify subgroups of patients. Hereby, patients could be divided into clusters that largely reflected the GOLD ABCD groups. By contrast, we could not reproduce the four clinical phenotypes identified by Burgel et al. in our cohort on the basis of a series of 8 variables that were essentially the same as those used by Burgel et al. [6] The addition of six new variables, air trapping, steps per day, QOL, satisfaction relationship, activities of daily living and fatigue, altered the clusters substantially and led to the formation of four clusters that were separated mainly by these behavioral parameters. We conclude that heterogeneity in the COPD population calls for a personalized medicine approach that is not based on the stratification of patients into subgroups but rather on individual characteristics.

## Supplementary Information

**Additional file 1: Table S1.** Cluster analysis (ABCD groups), using the variables exacerbation number and CAT. **Table S2.** Principal component analysis of clinical variables. **Table S3.** Principal component analysis behavior variables included.

## Data Availability

All data relevant to the study are included in the article or uploaded as supplementary information. The datasets used and/or analysed during the current study are available from the corresponding author on reasonable request.

## Abbreviations

GOLD: Global initiative for chronic obstructive lung disease
COPD: Chronic obstructive pulmonary disease
QOL: Quality of Life
mMRC: Modified Medical Research Council
CCQ: Clinical COPD Questionnaire
CAT: COPD Assessment Test
BMI: Body Mass Index
FEV_1_: Forced Expiratory Volume in 1 s
FVC: Forced Vital Capacity
PY: Packyears
IQR: Interquartile range
BDI-PC: Beck Depression Inventory for primary care
HAD: Hospital Anxiety and Depression
BDI-PC: Beck Depression Inventory-Primary Care
SGRQ: St. George’s Respiratory Questionnaire
BOD: Body mass index, airflow Obstruction and Dyspnea score
