# Supplementary material for: Stratification of COPD patients towards personalized medicine: reproduction and formation of clusters
Source: Respir Res. 2022 Dec 9;23:336. doi: 10.1186/s12931-022-02256-7 (PMC9733189; doi:10.1186/s12931-022-02256-7)
Supplement: Supplementary file 1 — Additional file 1: Table S1. Cluster analysis (ABCD groups), using the variables exacerbation number and CAT. Table S2. Principal component analysis of clinical variables. Table S3. Principal component analysis behavior variables included. [file 12931_2022_2256_MOESM1_ESM.docx]

Additional file 1

**Table S1. Cluster analysis (ABCD groups), using the variables exacerbation number and CAT**

|  | Group A | Group B | Group C | Group D |
| --- | --- | --- | --- | --- |
| Number | 15 | 51 | 2 | 39 |
| *Cluster numbers* | | | | |
| 1 | 0 *) | 0 | 0 | 21 (100) |
| 2 | 0 | 0 | 2 (100) | 18 (90) |
| 3 | 15 (100) | 0 | 0 | 0 |
| 4 | 0 | 51 (100) | 0 | 0 |
| *Variables used in clustering**)* | | | | |
| Exacerbations p/y | 0 [0-1] | 0 [0-1] | 3 [2-3] | 3 [2-3] |
| CAT | 7 [4-9] | 19 [15-23] | 2 [0-3] | 22 [16-26] |
| *Other patient and disease characteristics* | | | | |
| Male/ female % | 40/60 | 67/33 | 50/50 | 46/54 |
| Age in years | 63 [59-68] | 64 [56-68] | 68 [64-71] | 62 [55-69] |
| Smoking PY | 34 [20-45] | 44 [29-58] | 10 [0-20] | 32 [23-50] |
| FEV1 % pred | 49 [42-66] | 62 [51-74] | 70 [69-70] | 56 [35-68] |
| GOLD stage %  1  2  3  4 | 0  53  47  0 | 18  55  23  4 | 0  100  0  0 | 21  41  23  15 |
| BMI kg/m^2^ | 23 [21-25] | 29 [23-35] | 27 [25-29] | 26 [21-29] |
| CCQ total score | 0.7 [0.6-1.1] | 2.4 [1.6-2.8] | 0.6 [0.4-0.8] | 2.5 [1.8-3.3] |
| mMRC | 0 [0-1] | 1 [1-2] | 0.5 [0-1] | 1 [1-3] |
| BDI-PC total score | 1 [0-2] | 1 [1-4] | 0 [0-0] | 3 [1-6] |
| Steps per day | 6832 [5063-9522] | 4115 [2605-5723] | 8230 [2658-13801] | 5147 [4016-6772] |
| BOD score | 2 [1-3] | 3 [1-4] | 0.5 [0-1] | 3 [2-5] |
| NCSI quality of life | 10 [6-18] | 20 [10-32] | 3 [1-5] | 20 [12-34] |
| NCSI satisfaction relationship | 2 [2-3] | 3 [2-6] | 2 [2-2] | 3 [2-5] |
| NCSI activities of daily living | 7 [0-13] | 16 [5-25] | 3 [0-5] | 13 [8-27] |
| NCSI fatigue | 29 [26-35] | 41 [35-46] | 24 [9-38] | 44 [36-50] |

*) Data are presented as N (%) or median [25-75 interquartile], unless otherwise stated. PY: packyears, FEV1 % pred: Forced Expiratory Volume in 1 second percentage predicted, BMI: Body Mass Index, MMRC: Modified Medical Research Council, CCQ: Clinical COPD Questionnaire, BDI-PC: Beck Depression Inventory for primary care. *Definition of group A: CAT<10 and exacerbations <2, group B: CAT>=10 and exacerbations <2, group C: CAT<10 and exacerbations>=2, group D: CAT>=10 and exacerbations>=2

**) Hierarchical clustering is performed based on two variables: Exacerbation per/year and CAT.

**Table S2. Principal component analysis of clinical variables**

|  | **Principal component** | | | |
| --- | --- | --- | --- | --- |
|  | **PC1 *)** | **PC2 *)** | **PC3 *)** | **PC4 *)** |
| **Age** | -0.1487 | 0.6463 | -0.0719 | 0.3544 |
| **Smoking (PYs)** | 0.1966 | 0.2990 | -0.1077 | -0.6987 |
| **FEV1% pred** | -0.1512 | -0.2199 | 0.7503 | 0.1070 |
| **BMI** | -0.0467 | 0.4748 | 0.6053 | -0.1287 |
| **BDI-I-PC** | 0.4949 | -0.2667 | 0.2037 | -0.2378 |
| **mMRC** | 0.4832 | 0.3527 | -0.0004 | 0.1873 |
| **Exacerbations p/y** | 0.3525 | -0.1388 | -0.0255 | 0.5092 |
| **CCQ total** | 0.5582 | 0.0657 | 0.1084 | 0.0859 |

*) Contribution of principal component: resp. 26%, 17%, 15%, 13%.

**Table S3. Principal component analysis behavior variables included**

|  | **Principal component** | | | | |
| --- | --- | --- | --- | --- | --- |
|  | **PC1 *)** | **PC2 *)** | **PC3 *)** | **PC4 *)** | **PC5 *)** |
| **Age** | -0.1335 | 0.4328 | -0.1464 | 0.0452 | -0.0779 |
| **Smoked (PYs)** | 0.1165 | -0.0492 | -0.1913 | -0.5311 | 0.5024 |
| **FEV1% pred** | 0.0401 | 0.1079 | 0.6542 | 0.1215 | 0.0655 |
| **BMI** | -0.0251 | 0.4665 | 0.2403 | -0.2532 | 0.2315 |
| **BDI-I-PC** | 0.4480 | -0.1823 | 0.0788 | -0.1307 | -0.2345 |
| **mMRC** | 0.2813 | 0.3172 | -0.3111 | 0.0981 | -0.0024 |
| **Exacerbations p/y** | 0.2077 | -0.0783 | -0.1391 | 0.5359 | 0.1493 |
| **CCQ total** | 0.3377 | 0.1639 | -0.0066 | 0.0955 | 0.0743 |
| **Dynamic hyperinflation post** | 0.0093 | 0.3296 | 0.4517 | 0.1404 | -0.0365 |
| **Steps per day** | -0.1638 | -0.3649 | 0.1061 | 0.4228 | 0.3896 |
| **NCSI quality of life** | 0.4620 | -0.1795 | 0.1323 | -0.1003 | -0.1467 |
| **NCSI satisfaction relationship** | 0.3685 | -0.1956 | 0.2317 | -0.1356 | -0.0147 |
| **NCSI activities of daily living** | 0.2944 | 0.2999 | -0.2173 | 0.2753 | -0.1457 |
| **NCSI fatigue** | 0.2579 | 0.1092 | -0.0133 | 0.1058 | 0.6362 |

*) Contribution of principal component: resp. 26%, 14%, 11%, 10%, 7.5%.
